# A Comparison of the Glasgow Coma Scale Score with Full Outline of Unresponsiveness Scale to Predict Patients' Traumatic Brain Injury Outcomes in Intensive Care Units

**DOI:** 10.1155/2014/289803

**Published:** 2014-06-10

**Authors:** Rostam Jalali, Mansour Rezaei

**Affiliations:** Kermanshah University of Medical Sciences, Kermanshah 67189-74589, Iran

## Abstract

*Background*. Neurological assessment is an essential element of early warning scores used to recognize critically ill patients. We compared the performance of the Glasgow Coma Scale (GCS) with Full Outline of Unresponsiveness (FOUR) scale as an alternative method in the identification of clinically relevant outcomes in traumatic brain injury. *Objective*. The purpose of this study was to compare the performance of GCS with FOUR scale. *Methods*. For this study 104 patients with brain injury were recruited from the ICU of Taleghani Hospital, a major teaching hospital in Kermanshah in the western part of Iran. Data was collected concurrently from the ICU admissions by three well-educated nurses and then checked for accuracy by the researcher. Patients were followed up until two weeks or hospital discharge to record their survival status. As a final point expected risk of mortality was calculated using the original formulas for each scale. *Results*. The mean age of 104 participants was 41.38 ± 18.22 (rang 17 to 86 years) mostly (81 patients 77.9%) males. The FOUR scale has a better prediction for death than GCS. *Conclusion*. It appears that FOUR scale had better predictive power for mortality and may be a suitable alternative or complementary tool for GCS.

## 1. Introduction

Traumatic brain injury (TBI) is one of the major causes of death and severe disability worldwide. In the USA alone, this type of injury causes 290,000 hospital admissions, 51,000 deaths, and 80,000 permanently disabled survivors [1]. The centers for disease control (CDC) and prevention analysis estimated that approximately 1.4 million people are hospitalized each year for medical care related to traumatic brain injury [2], and the average cost per ICU stay was US$ 4846 ± 5084. Costs per life saved and per life-year saved were US$ 9533 and US$ 313.60, respectively [3]. There has been growing interest in scoring systems for measuring the severity of illness and predicting outcomes in critically ill patients since 1974 [4]. Reliable outcome prediction at the acute stage in the neuro-ICUs is thus important [5]. The most widely used and most studied coma scale to date is the Glasgow Coma Scale (GCS). The GCS was broadly accepted as an instrument to classify the severity of TBI because it was easy to use and reproducible [1]. The GCS in the appendix was adopted to enhance communication among practitioners by providing a common language for assessing the depth and duration of impaired consciousness and coma [6]. Since then it has become the gold standard against which newer scales are compared. Despite its widespread use, the GCS has some significant limitations, including variations in interrater reliability and predictive validity [7]. Other shortcomings of the GCS are inability of verbal component testing in intubated patients, inability to grade breathing pattern and brainstem reflexes, and inability to detect subtle change in neurological examination. However, several ICU scoring systems have been developed to overcome perceived deficiencies in the GCS. The Full Outline of Unresponsiveness (FOUR) score, a newer coma scale in the appendix developed in the Mayo Clinic, evaluates 4 components: eye, motor responses, brainstem reflexes, and respiration [4].

In this study, based on GCS deficiencies, we hypothesized that GCS might be ineffective for the initial assessment of traumatic brain injury and a simple scoring system such as the FOUR scale might demonstrate similar test performance. Therefore, we compared the test performance of GCS with FOUR scale as an alternative tool in the prediction of outcomes in traumatic brain injury.

## 2. Materials and Methods

We prospectively studied the FOUR scale in 104 patients with brain injury in an intensive care unit of the major teaching hospital of Kermanshah (Taleghani Hospital), west of Iran. The ICU consists of 12 beds and data was collected from January 2007 to February 2008. All data were collected concurrently from ICU admissions. Patients aged ≥16 were eligible for enrollment. We excluded patients whose eye, verbal, or motor GCS components were not identifiable. Spinal cord injury and surgical patients were excluded. Patients were excluded if they were heavily sedated or receiving neuromuscular function blockers. We had to exclude 16 patients because they were heavily sedated in this early period of head injury, and thus we were unable to obtain FOUR or GCS accurately. Patients with multiple ICU admissions, only data from the first admission was collected. For each patient's demographic data was collected. We recorded first day GCS and FOUR, respectively. The expected risk of death was calculated using the original formulas of each severity scoring system. The cutoff points for the GCS scale were scores under or equivalent to five (GCS ≤ 5) and for FOUR were six or lower (FOUR ≤ 6). Three nurses tested the FOUR score and the GCS. Each of the nurses had more than 5 years of clinical experience in a neurological/neurosurgical intensive care unit (ICU) and was reinstructed in GCS and the FOUR score. Subsequently, raters were provided with a one-page handout written instruction describing both the FOUR score and the GCS and were asked to grade a few patients using both the GCS and the FOUR score scale. Interrater reliabilities for FOUR score and GCS were 0.98 and 0.96, respectively. Patients were followed up until two weeks or hospital discharge in order to record their survival status. All patients were prospectively enrolled and provided informed consent by their guardian. Comparison of the discrepancies between the scales was undertaken by cross-tabulating their prediction at a fixed decision criterion. The observed and expected numbers of deaths within each stratum were compared and their sensitivity, specificity, and accuracy were statistically evaluated by the Youden index (Table 1). For data entry and analysis, SPSS 14 was employed. Both descriptive (mean, SD, and frequency) and inferential statistics test included the Yoden index, and diagnostic values (TP, TN, FP, and FN) were used.

## 3. Results

In this study 104 patients, 23 (22.15%) females and 81 (77.9%) males, with mean age of 41.38 ± 18.22 (from 17 to 86 years) were studied. Sensitivity of both scales was 68.4% (Table 2). GCS predicted 26 deaths accurately. The agreement between GCS and patients outcome was 30% and between FOUR scale and patients outcome was 44.9%. This agreement between two scales was 43.8% (Table 3). No relationship between sex and age was found in this regard. The Youden index showed that FOUR scale (45.7%) has a better prediction for death than GCS (32.0%). Furthermore, Kappa agreement coefficient for agreement between FOUR (*P* = 0.006) and GCS (*P* = 0.016) with patient's outcome was statistically significant (Table 3). Means of scores in dead and alive patients for GCS were 4.62 ± 2.094 and 6.58 ± 2.281, and for FOUR they were 4.7 ± 3.471 and 8.42 ± 2.925, respectively. *t*-test showed a significant difference between means of the alive and dead subjects in both scales (*P* < 0.0001).

## 4. Discussion

The FOUR score is simple to use, includes the minimal necessities of neurological testing in impaired consciousness, and specifically recognizes certain unconscious states [4]. This new coma scale includes important clinical neurological findings in patients with impaired consciousness [8] and this study shows that it can be assessed by ICUs nurses. Furthermore, this study confirmed previous studies that the FOUR score is a robust predictor of in-hospital mortality, functional outcome at hospital discharge, and overall survival in patients seen for neurologic complaints [4, 9].

The results of this study showed that FOUR scale is better than GCS. These findings confirmed Ledoux study which showed that FOUR score had better prediction than previous scale for classifying and communicating impaired consciousness [10], in emergency department [11, 12], after cardiac arrest [13], and in intensive care units [14]. Compared with the GCS, this new coma scale does not depend on a verbal response and provides greater neurological detail by inclusion of brainstem reflexes and breathing patterns [15, 16]. The present study is one of the first validations of the FOUR score in the ICU outside the institution that developed the FOUR score. Furthermore, for further validation of the FOUR scale by intensive care nurses, the results of the study revealed that the FOUR score provides more neurologic information than GCS and thus the FOUR score can be used by every ICU nurse [17]. However, in one study, the FOUR score is a valid tool with good interrater reliability that is comparable to the GCS in predicting outcome [18]. It offers the advantage of being performable in intubated patients and of identifying nonverbal signs of consciousness by assessing visual pursuit, and hence minimal signs of consciousness [19], but in contrast with the study to assess the value of the two scoring systems in prediction outcome, the results revealed that theFOUR score is not superior to the GCS [18]. Also, in one study, it was found that the small advantage in interrater reliability of the FOUR score is most likely insufficient to replace the GCS, a score with a long tradition in intensive care [15], and in another study it was found that the GCS scale was one of the best predictors of mortality in emergency medical admissions [2]. Finally, studies showed that none of the simpler scores should replace GCS for the formal evaluation of a critically ill patient [20, 21].

There are some limitations in this study. The sample may not have covered enough severely injured patients. GCS and FOUR scores were determined within 24 h of admission to the ICU by only one investigator. Another limitation was that the target enrollment cohort was not reached, and approximately 35% of the studied patient population included alert patients. This increases the chance of interobserver agreement because no neurologic abnormality will have to be identified. A study of a larger group of stuporous or comatose patients would be desirable. This was a single center study, so the generalizability to other ICUs has not been proved yet.

## 5. Conclusion

Although, within 40 years since its introduction, the GCS has remained the cornerstone of initial traumatic brain injury evaluation by out-of-hospital personnel, emergency physicians, trauma surgeons, and neurosurgeons, on the basis of the findings of this study and considering the results of the previous studies, the FOUR score appears to be an easier tool to use and it provides a more comprehensive neurological assessment. In modern ICUs, multiple scores are repetitively used. Ideally, these scores should be simple, reliable, and predictive for relevant outcomes and/or relevant clinical decisions. The widespread adoption of such a tool may enhance the ability to accurately predict survivability, impacting the treatment and management of these patients and their families.

## Figures and Tables

**Table 1:** Youden index (*J* = 1 − (*α* + *β*)) for explaining reveals and results interpretation.

Observed	Prediction	
	Dead	Alive
Dead	1 − *β*	*β*
Alive	*α*	1 − *α*

**Table 2:** Diagnostic values of GCS and FOUR for prediction of death.

Scale prediction power	GCS (%)	FOUR (%)
Sensitivity	68.4	68.4
Specificity	63.6	77.3
Positive predictive value	52.0	63.4
Negative predictive value	77.8	81.0
Accuracy	65.4	74.0

**Table 3:** Agreement between two scales for patient's outcome: no (%).

Scales	Prediction	Outcome		Total	*P* value
		Alive	Dead		
FOUR	Alive	51 (77.3)	12 (31.6)	63 (60.6)	0.006
	Dead	15 (22.7)	26 (68.4)	41 (39.4)	

GCS	Alive	42 (63.6)	12 (31.6)	54 (51.9)	0.016
	Dead	24 (36.4)	26 (68.4)	50 (48.1)	

Total		66 (100)	38 (100)	104 (100)	

## Appendix

### A. Glasgow Coma Scale (GCS) and Full Outline of Unresponsiveness (FOUR)

#### A.1. Glasgow Coma Scale (GCS)

##### A.1.1. Eye Response

One has the following:

4 = eyes open spontaneously
3 = eye opening to verbal command
2 = eye opening to pain
1 = no eye opening.

##### A.1.2. Motor Response

One has the following

6 = obeying commands
5 = localizing pain
4 = withdrawal from pain
3 = flexion response to pain
2 = extension response to pain
1 = no motor response.

##### A.1.3. Verbal Response

One has the following

5 = oriented
4 = confused
3 = inappropriate words
2 = incomprehensible sounds
1 = no verbal response.

#### A.2. Full Outline of Unresponsiveness (FOUR)

##### A.2.1. Eye Response

One has the following:

4 = eyelids open or opened, tracking, or blinking to command
3 = eyelids open but not to tracking
2 = eyelids closed but open to loud voice
1 = eyelids closed but open to pain
0 = eyelids remaining closed with pain stimuli.

##### A.2.2. Motor Response

One has the following:

4 = thumbs up, fist, or peace sign
3 = localizing to pain
2 = flexion response to pain
1 = extension response
0 = no response to pain or generalized myoclonus status.

##### A.2.3. Brain Stem Reflexes

One has the following:

4 = pupil and corneal reflexes present
3 = one pupil wide and fixed
2 = pupil or corneal reflexes absent
1 = pupil and corneal reflexes absent
0 = absent pupil, corneal, or cough reflex.

##### A.2.4. Respiration

One has the following:

4 = regular breathing pattern
3 = Cheyne-stokes breathing pattern
2 = irregular breathing
1 = triggering ventilator or breathing above ventilator rate
0 = apnea or breathes at ventilator rate.
